# Cyathostomine egg reappearance period following ivermectin treatment in a cohort of UK Thoroughbreds

**DOI:** 10.1186/s13071-018-2638-6

**Published:** 2018-01-25

**Authors:** Rebecca A. Molena, Laura E. Peachey, Angela Di Cesare, Donato Traversa, Cinzia Cantacessi

**Affiliations:** 1 Department of Veterinary Medicine, University of Cambridge, Cambridge, UK; 2 Faculty of Veterinary Medicine, University of Teramo, Località Piano d’Accio, Teramo, Italy

**Keywords:** Cyathostominae, Ivermectin, Anthelmintic resistance, Macrocyclic lactones, Egg reappearance period, Reverse line blot

## Abstract

**Background:**

In spite of the emergence of populations of drug-resistant cyathostomines worldwide, little is known of parasite species responsible for ‘early egg shedding’ in cohorts of horses subjected to treatment with widely used anthelmintics, e.g. ivermectin (IVM). In this study, we determined the cyathostomine egg reappearance period (ERP) after IVM treatment in a cohort of yearlings from a large Thoroughbred (TB) stud farm in the United Kingdom, and identified species of cyathostomines with reduced ERP using a combination of fundamental parasitology techniques coupled with advanced molecular tools.

**Methods:**

Individual faecal samples were collected from TB yearlings with cyathostomine infection prior to IVM treatment, as well as at 14, 21, 28, 35, 42 and 49 days post-treatment. Faecal egg counts (FEC) were performed for each individual sample for determination of ERPs. In addition, individual larval cultures were performed and representative numbers of third-stage larvae (L3s) harvested from each culture were subjected to molecular species identification via PCR-Reverse Line Blot (RLB).

**Results:**

Prior to IVM treatment, 11 cyathostomine species were detected in faecal samples from TB horses enrolled in this study, i.e. *Cyathostomum catinatum*, *Cylicostephanus longibursatus*, *Cylicostephanus goldi*, *Cylicocyclus nassatus*, *Cylicostephanus calicatus*, *Cyathostomum pateratum*, *Cylicocyclus radiatus*, *Paraposteriostomum mettami*, *Coronocyclus labratus*, *Cylicocyclus insigne* and *Cylicocyclus radiatus* variant A. Of these, eggs of *Cya. catinatum*, *Cys. longibursatus*, *Cyc. nassatus* and *Cyc. radiatus* could be detected at 28 days post-treatment, while from day 42 onwards, cyathostomine species composition reflected data obtained pre-IVM treatment, with the exception of eggs of *Cor. labratus* and *Cyc. insigne* which could no longer be detected post-IVM administration.

**Conclusions:**

This study provides valuable data on the occurrence of IVM-resistance in cyathostomines in the UK. Nevertheless, further investigations are needed to shed light on the prevalence and incidence of drug-resistance in this country, as well as other areas of the world where equine trade is substantial.

**Electronic supplementary material:**

The online version of this article (10.1186/s13071-018-2638-6) contains supplementary material, which is available to authorized users.

## Background

Intestinal nematodes of the subfamily Cyathostominae (family Strongylidae), also known as ‘cyathostomins’ or ‘small strongyles’ are ubiquitous parasites of equines, with reported prevalences of up to 100% in many regions of the world, including Europe and North America [1–4]. The subfamily currently includes 52 recognised species [5–7], characterised by a direct (oro-faecal), non-migratory life-cycle. Adult males and females live in the lumen of the large intestine, where the latter shed eggs that are excreted in the environment with the host faeces; under suitable conditions of temperature and humidity, first-stage larvae (L1s) hatch from the eggs and develop through to second- (L2s) and third-stage larvae (L3s, the infective stages) [8]. These are ingested by susceptible equine hosts while grazing, and move to the large intestine where they become encysted within the mucosal layer and mature to fourth-stage larvae (L4s) [9]. Alternatively, within the mucosa, L3s can undergo hypobiosis and survive up to 2.5 years before resuming their development [10]. Subsequently, L4s emerge from the cysts and migrate to the intestinal lumen, where they undergo their final development to adult males and females [11]. The pre-patent period ranges from 5 to 12 weeks depending on parasite species [12].

While infections by cyathostomines often remain subclinical, clinical signs associated with heavy parasite burdens may occur, particularly in young, geriatric and immunocompromised equines [13, 14]. In particular, the synchronous emergence of L4s into the intestinal lumen may be accompanied by a protein losing enteropathy characterized by a sudden onset of diarrhoea, weight loss and dehydration (‘larval cyathostominosis’), that can prove fatal in 50% of cases [15–17]. Additionally, non-specific weight loss, non-strangulating infarction, tympanic colic and mild non-specific colic have been observed in horses infected by large numbers of worms [13, 18–23].

Traditionally, cyathostomine infections are controlled via the regular administration of parasiticides, i.e. ‘anthelmintics.’ Currently licenced anthelmintics with efficacy against cyathostomines include the benzimidazoles (BZ), e.g. fenbendazole (FBZ) [24, 25], the tetrahydropyrimidines (THP), e.g. pyrantel pamoate (PYR) [26, 27] and the macrocyclic lactones (ML), e.g. ivermectin (IVM) and moxidectin (MOX) [28–30]. However, the widespread and indiscriminate use of these chemotherapeutics has led to the emergence of populations of cyathostomines resistant to all of these drugs [31–34]. In particular, anthelmintic resistance (AR) of cyathostomine populations to BZs is widespread [35–40], whilst resistance to PYR is common in some regions [37–39, 41–43]. Therefore, given that none of the novel anthelmintics used in other veterinary species developed over the last decade are licenced for use in horses [44], current deworming programs aimed to control cyathostomine infections rely upon ML compounds. Amongst these, IVM and MOX are used interchangeably; nevertheless, the relative high cost of MOX compared to IVM [45], as well as ongoing efforts to preserve its efficacy against encysted larvae (by avoiding its excessive use) [36, 46], make IVM the most widely used anthelmintic against cyathostomine infections.

Regrettably, the widespread use of ML has been accompanied by reports of emerging AR in cyathostomine populations globally, primarily evidenced by a reduction in egg reappearance period (ERP) (i.e. the time between administration of anthelmintics and the detection of parasite eggs in faeces) [38, 42, 47–53]. Reduced ERPs have been associated with the survival of luminal L4 stages, that reach sexual maturity before encysted L4s and/or newly ingested L3s [54]. However, in spite of these reports, little evidence of AR to IVM in cyathostomine populations based on faecal egg count reduction test (FECRT) analyses is available [47, 51, 52]. Further investigations are therefore needed to better understand the occurrence of AR to IVM in cyathostomine populations, and to design strategies to prevent and/or mitigate its spread. In order to achieve this outcome, knowledge of the fundamental biology of cyathostomines, and in particular of species responsible for ‘early egg shedding’, is necessary. Thus far, only four studies have provided data on species of cyathostomines responsible for reduced ERPs after IVM and MOX treatment in Europe [55, 56] and the USA [57, 58]. Although the findings from these studies were largely similar, some differences were observed, likely due to variations in cyathostomine species epidemiology between geographical locations. Additional studies conducted in a range of countries, characterised by different epidemiologies of cyathostomine infections, may help elucidate these issues. In particular, given the substantial contribution that the Thoroughbred (TB) racehorse industry provides to the economy of the United Kingdom, AR is of particular concern. In this study, we determined the ERP after IVM treatment in a cohort of yearlings from a large TB stud farm in the UK and identified species of IVM-‘resistant’ cyathostomines using a combination of fundamental parasitology techniques coupled with advanced molecular tools.

## Methods

### Sample collection

Sample collection was performed between April and June 2017. A cohort of 54 TB yearlings housed in a stud farm in the south-east of England was initially screened for this study. In particular, all yearlings had been treated with FBZ (Panacur 18.75% FBZ, MSD, Milton Keynes, UK) and IVM (Eqvalan, 1.87% IVM, Merial, Harlow, UK) approximately 10 weeks prior to the beginning of the study, and with praziquantel (PZQ) (Equitape, 90 mg/g PZQ, Zoetis, Tadworth, UK) 3 weeks prior to the start of the study, respectively. On Day 0 (D0), freshly-voided faecal samples were collected from individual horses and subsequently screened for infections by parasitic nematodes using a centrifugal floatation faecal egg count (FEC) technique sensitive to 1 egg per gram (EPG) [59]. Samples were also screened for tapeworm infections (by *Anoplocephala* spp.) using a standard double sugar flotation technique [60]. Immediately after sampling, all animals were treated with 0.2 mg/kg of IVM (Eqvalan®, Merial, Harlow, UK). Horses with FEC > 75 strongyle EPG were selected for the study. From these, additional faecal samples were collected weekly up to 7 weeks post-IVM treatment and subjected to faecal examination as described above.

### Faecal egg count reduction test (FECRT) and determination of egg reappearance period (ERP)

Arithmetic means of FEC values obtained from individual faecal samples at D0 and Day 14 (D14) were used to estimate faecal egg count reduction (FECR), using guidelines established by the World Association for Advancement of Veterinary Parasitology [61], according to the formula:

$$ FECR\%=\frac{EPG\ \left( pre\hbox{-} treatment\right)- EPG\ \left( post\hbox{-} treatment\right)}{EPG\ \left( pre\hbox{-} treatment\right)}\times 100 $$

A FECR of < 95% with 95% lower confidence limits (LCL) was considered to indicate potential AR, according to previously published recommendations [62, 63]. In this study, the ERP was defined as the first time point (post-IVM treatment) at which a mean FEC that exceeded 10% of the mean FEC at D0 was observed [42, 64]. Statistical analyses were performed using Microsoft Office Excel 2013.

### Larval culture and harvest of cyathostomine larvae

In order to identify species of cyathostomines with reduced ERP post-IVM treatment, faecal samples containing cyathostomine eggs were subjected to larval culture as described by van Doorn et al. [55]. Briefly, individual faecal samples were placed in open trays at room temperature for 14 days. Following incubation, L3s were collected using the Baermann’s apparatus, washed three times with distilled water, centrifuged at 14,000× *rpm* for 5 min and re-suspended in 1 ml 100% ethanol before storing at -20 °C.

### Nucleic acid extraction and polymerase chain reaction-reverse line blot (PCR-RLB)

From each faecal sample, four representative pools, each containing 10 larvae, were prepared according to the method described by Kooyman et al. [56]; this method has been proven accurate for the semi-quantitative determination of cyathostomine species composition in a given sample [56]. Briefly, aliquots of L3s from each sample were placed on individual Petri dishes and observed under a stereomicroscope; for each of these, 40 larvae were picked using a wide orifice tip and transferred to four 1.5 ml centrifuge tubes (10 larvae in each tube). 73 μl of proteinase K was added to 1 ml of Worm Lysis Buffer (WLB) (50 mM KCl, 10 mM tris pH 8.3, 2.5 mM MgCl_2_, 0.45% NP-40, 0.45% tween-20 and 0.01% gelatin; ThermoFisher Scientific, Waltham, MA, USA; Sigma-Aldrich, St. Louis, MO, USA) [65] and 50 μl of Proteinase K/WLB solution was added to each L3 pool. Pools were incubated overnight at 56 °C. Proteinase K was then inactivated by incubation at 95 °C for 15 min; lysates were stored at -20 °C until further processing.

Identification of the species of cyathostomines present in each pool was performed using an established PCR-RLB hybridisation method [66] with slight modifications [67]. Briefly, genomic DNA extracted from each pool of L3s was subjected to nested-PCR amplification of the intergenic spacer (IGS) region using biotin labelled primers [66]. The PCR products were then incubated with Biodyne C membrane bound specific DNA probes, using a Miniblotter 45 (Bioworld), for 21 different cyathostomine species and two conserved probes for the genus *Strongylus* and the subfamily Cyathostominae [66, 67], incubated with extravidin peroxidase (Sigma-Aldrich, St. Louis, MO, USA) and visualised using chemiluminescence detection.

## Results

**Fig. 1:**
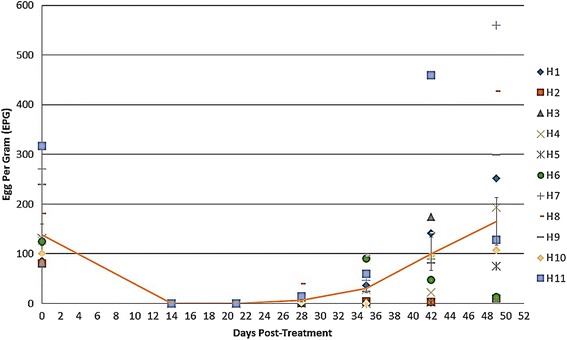
Mean faecal egg counts (FEC) recorded for each horse (H) enrolled in the study, prior to ivermectin administration (D0), as well as at D14, D21, D28, D35, D42 and D49, following anthelmintic treatment

Of the 54 yearlings screened at D0, 11 had FEC > 75 EPG and were therefore enrolled in this study (Additional file 1: Table S1). Strongyle FEC performed from samples collected from individual horses at D0 (pre-IVM treatment), and at D14, D28, D35, D42 and D49 post-IVM treatment, as well as corresponding means, are shown in Additional file 1: Table S1 and Fig. 1. At D14, a 100% FECR was observed in all 11 horses enrolled in the study, whereas the numbers of strongyle eggs in individual faecal samples exceeded the set threshold for ERP at D28 in Horse 8, D35 in Horses 1, 4, 6, 7, 9, and 11, D42 in Horses 3 and 10 and D49 in Horses 2 and 5 (Additional file 1: Table S1 and Fig. 1).

**Fig. 2:**
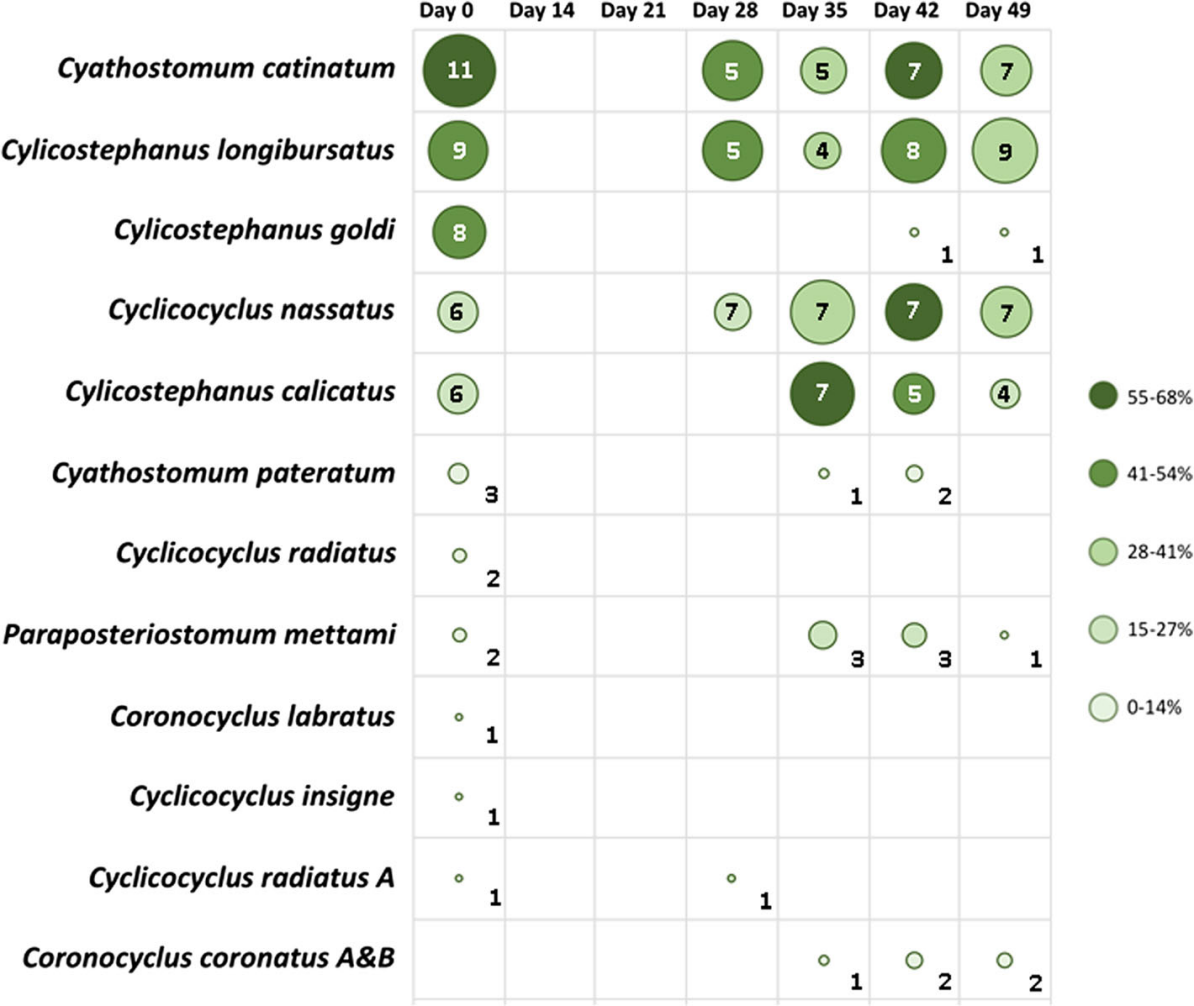
Species of cyathostomine identified from larval cultures of horses enrolled in this study, prior to (D0) and following ivermectin administration (D14, D21, D28, D35, D42 and D49, respectively). Bubble sizes correspond to the relative proportions of horses infected with the corresponding species, while the exact number (out of a total of 11 horses enrolled in the study) is indicated within the circle/cell. Shades of green indicate the percentage of larval pools (see Methods) from which the corresponding species was identified

Prior to IVM treatment, larval culture of individual samples collected from horses enrolled in this study, coupled with PCR-RLB for species identification, revealed infections by 11 cyathostomine species. In decreasing order of frequency of detection per horse, these were *Cyathostomum catinatum*, *Cylicostephanus longibursatus*, *Cylicostephanus goldi*, *Cylicocyclus nassatus*, *Cylicostephanus calicatus*, *Cyathostomum pateratum*, *Cylicocyclus radiatus*, *Paraposteriostomum mettami*, *Coronocyclus labratus*, *Cylicocyclus insigne* and *Cylicocyclus radiatus* variant A (Fig. 2). One additional species, *Coronocyclus coronatus*, was detected post-IVM treatment at D35, D42 and D49 (Fig. 2). Species of cyathostomines detected in faecal samples from individual horses post-IVM treatment via RLB are listed in Fig. 2. The first species detected post-treatment, at D28, were *Cya. catinatum* (in five horses), *Cys. longibursatus* (in five horses), *Cyc. nassatus* (in three horses) and *Cyc. radiatus* (in one horse) (Fig. 2). At Day 35, a total of 7 species were detected in a total of 8 horses, i.e. those detected at D28 (except *Cyc. radiatus* variant A) together with *Cys. calicatus*, *Cya. pateratum*, *P. mettami* and *Cor. coronatus*; in particular, the latter was not detected amongst species identified pre-IVM treatment (Fig. 2). Eggs of *Cys. goldi* reappeared in one horse from D42, while those of *Cor. labratus* and *Cyc. insigne* were no longer detected post-IVM treatment (Fig. 2). From D42 onwards, cyathostomine species composition reflected data obtained pre-IVM treatment (Fig. 2).

## Discussion

The acquisition of comprehensive data on the occurrence and prevalence of potential drug-resistant species of cyathostomine is the necessary basis on which to build studies aimed to unravel the fundamental molecular biology of AR in this complex group of nematodes, as well as to develop and test new chemotherapeutics and/or alternative strategies for parasite control. In this study, we aimed to determine the ERP of cyathostomines following treatment with IVM, and to identify the species responsible for ‘early egg shedding’. Specifically, the ERP was defined as the earliest time point post-IVM treatment in which the number of EPG obtained following FEC analysis of samples collected from individual horses was ≥ 10% of the corresponding number observed pre-anthelmintic treatment [42, 64]. This definition was selected to minimise errors associated with the sensitivity of the FEC technique used that, although reported to be as low as 1 EPG [59], does not allow to unequivocally rule-out false negative results.

Data obtained from the FECRT suggest that the administration of IVM was effective in eliminating adult populations of cyathostomines 14 days post-treatment; nevertheless, the ERP was shorter than that originally reported for an IVM-susceptible population of parasites (i.e. 8–13 weeks) [68, 69]. Whilst 9 out of 11 horses had detectable strongyle eggs at D28, FECs in all of these horses but one were below 10% of the FEC recorded pre-treatment, thus failing to reach the set threshold of ERP at this time-point. In seven of these horses, FECs above the ERP threshold were subsequently observed at D35 post-IVM treatment. This is in agreement with the majority of recent reports from Europe and the USA, in which strongyle eggs were detected from 28 days post-IVM treatment [52, 55, 57, 70, 71]. This data suggests that populations of IVM-resistant cyathostomines are indeed developing in the TB stud farm where the investigation was carried out, and that overt AR will likely emerge in future, should the current regime of IVM administration continue. Indeed, it must be pointed out that, in the stud farm under consideration, IVM and MOX are regularly administered (in rotation) to horses with > 50 EPG. While aimed to provide ‘targeted’ treatments to horses with relatively high infection intensity, this practice may facilitate the emergence of AR by preventing the development and maintenance of refugia of susceptible parasites [72]. Thus, a threshold of > 200 EPG for treatment administration, combined with strategies of environmental control aimed to reduce the numbers of free-living larvae on the pasture (e.g. by bi-weekly removal of faecal matter; cf. [73, 74]) may assist in slowing the process of developing AR in these parasite populations [74, 75].

Prior to IVM-treatment, the most prevalent species of cyathostomines in the TB population under investigation were, in decreasing order of prevalence, *Cya. catinatum*, *Cys. longibursatus*, *Cys. goldi* and *Cyc. nassatus*. With the exception of *Cys. goldi*, whose eggs could not be detected until D42 post-IVM treatment, eggs from these species were observed at D28 in 10 out of 11 horses, albeit the corresponding FEC did not reach the set ERP threshold. These findings are supported by data from earlier studies that report cyathostomine species with a relatively high prevalence pre-IVM and MOX administration to display a reduced ERP of 4–5 weeks post-IVM, 4 weeks post-MOX [57, 58, 76] and ‘anthelmintic resistance’ [54]. One possible explanation is technical, and linked to the high relative proportion of these species in faecal samples from horses pre- and post-anthelmintic treatment; indeed, given that species identification via the RLB technique used in this study relies on a randomly selected (representative) sub-population of L3s, the likelihood of eggs from a given species to be successfully identified is directly correlated to its initial prevalence in the sample under consideration. In contrast to this hypothesis, van Doorn et al. [55] reported that, regardless of species prevalence prior to treatment, eggs of *Cylicocyclus* spp. were consistently detected prior to those of other species post-IVM treatment [55]. Interestingly, in spite of the relatively high prevalence of *Cys. goldi* in horses pre-treatment here (i.e. 6/11), eggs of this species could not be detected in faecal samples until D42 post-IVM treatment, thus indicating greater susceptibility of this species to IVM. This observation contrasts findings from Ionita et al. [57], that reported a (post-IVM) ERP for *Cys. goldi* of 5 weeks. Given that the molecular events that determine the emergence of AR in cyathostomines are, thus far, poorly understood (with mutations of the α-subunit gene of a GluCl channel [77, 78], reduction in drug uptake [79, 80] and overexpression of parasite P-glycoproteins (P-gps) [81, 82], all proposed as potential underlying mechanisms), the identification of susceptible (e.g. *Cys. goldi*) and resistant species of cyathostomines (e.g. *Cya. catinatum*, *Cys. longibursatus*), may provide a suitable platform for future studies aimed to investigate the molecular basis of cyathostomine AR, at the DNA, mRNA and protein level.

At D42 and D49 post-IVM treatment, the cyathostomine species composition observed in faecal samples via RLB largely reflected that of pre-IVM, with the exception of *Cor. labratus* and *Cyc. insigne*, whose eggs were no longer detected post-anthelmintic administration. In addition, at D35, eggs of *Cor. coronatus* were detected in one horse; this species had not been identified from samples pre-IVM. The low prevalence of *Cor. labratus* and *Cyc. insigne* species pre-IVM administration (1/11 horses), as well as the likely low proportion of *Cor. coronatus* in samples pre-treatment, may have led to L3s of these species not being selected amongst those that underwent PCR-RLB screening (see also [83]). Indeed, while this method represents a faster and relatively inexpensive alternative to sequencing of DNA amplicons from individual larvae (which is unfeasible under natural conditions of infection, when hundreds to thousands of larvae can develop in faecal samples from horses with heavy parasite burdens), its intrinsic limitation consists in the inability to unequivocally rule out the presence of other, less represented species of cyathostomines in the initial faecal sample. Nevertheless, in the future, the study of the ‘nemabiome’ (i.e. the characterisation of whole parasite communities within a given sample via high-throughput amplification and sequencing of nematode genetic loci [84]) of faecal samples from horses prior to and post-anthelmintic treatment, will provide the scientific community with means to overcome this constraint.

## Conclusions

While this study provides valuable data on the occurrence of IVM-resistance in the UK, further investigations are needed to shed light on the prevalence and incidence of drug-resistance in this country, as well as other areas of the world where equine trade is substantial. Primarily, it should be established whether our observation that the most prevalent species of cyathostomines are responsible for shortened ERPs, are consistent across different geographical areas, or whether emergence of AR is dependent on specific micro-climatic and/or epidemiological conditions. This information is indeed crucial to inform future strategies aimed to mitigate the occurrence and spread of AR.

## Additional file

Additional file 1: Table S1. Raw faecal egg count (FEC) data collected from individual horses enrolled in this study, prior to ivermectin (IVM)-treatment, as well as at Day (D) 14, 21, 28, 35, 42 and 49 post-treatment. (DOCX 25 kb)

## Abbreviations

AR: Anthelmintic resistance
BZ: Benzimidazole
*Cya.*: *Cyathostomum*
*Cyc.*: *Cylicocyclus*
*Cys.*: *Cylicostephanus*
EPG: Eggs per gram
ERP: Egg reappearance period
FBZ: Fenbendazole
FEC: Faecal egg count
FECR: Faecal egg count reduction
FECRT: Faecal egg count reduction test
IVM: Ivermectin
ML: Macrocyclic lactones
MOX: Moxidectin
PYR: Pyrantel
PZQ: Praziquantel
RLB: Reverse Line Blot
TB: Thoroughbred
THP: Tetrahydropyrimidines
WLB: Worm Lysis Buffer
