# Supplementary material for: Cyathostomine egg reappearance period following ivermectin treatment in a cohort of UK Thoroughbreds
Source: Parasit Vectors. 2018 Jan 25;11:61. doi: 10.1186/s13071-018-2638-6 (PMC5785887; doi:10.1186/s13071-018-2638-6)
Supplement: Additional file 1: Table S1. — Raw faecal egg count (FEC) data collected from individual horses enrolled in this study, prior to ivermectin (IVM)-treatment, as well as at Day (D) 14, 21, 28, 35, 42 and 49 post-treatment. (DOCX 25 kb) [file 13071_2018_2638_MOESM1_ESM.docx]

**Additional file 1: Table S1.** Raw faecal egg count (FEC) data collected from individual horses enrolled in this study, prior to ivermectin (IVM)-treatment, as well as at Day (D) 14, 21, 28, 35, 42 and 49 post-treatment.

| Horse | D0 | D14 | D21 | D28 | D35 | D42 | D49 |
| --- | --- | --- | --- | --- | --- | --- | --- |
| H1 | 86.5 | 0 | 0 | 5 | 36 | 141 | 252 |
| H2 | 80 | 0 | 0 | 1 | 4 | 3 | 10 |
| H3 | 81 | 0 | 0 | 2 | 3 | 174 | 9 |
| H4 | 126 | 0 | 0 | 3 | 28 | 22 | 193 |
| H5 | 131 | 0 | 0 | 0 | 0 | 2 | 75 |
| H6 | 125 | 0 | 0 | 7 | 91 | 47 | 13 |
| H7 | 271 | 0 | 0 | 3 | 46 | 89 | 560 |
| H8 | 181 | 0 | 0 | 40 | 95 | 142 | 427 |
| H9 | 239 | 0 | 0 | 0 | 24 | 82 | 299 |
| H10 | 100 | 0 | 0 | 2 | 0 | 96 | 107 |
| H11 | 317 | 0 | 0 | 14 | 59 | 459 | 127 |
